# Establishment of a piglet model for peritoneal metastasis of ovarian cancer

**DOI:** 10.1186/s12967-022-03533-1

**Published:** 2022-07-21

**Authors:** Eun Ji Lee, Soo Jin Park, Aeran Seol, Hyunji Lim, Sumin Park, Ji Yeon Ahn, Jeong Mook Lim, Hee Seung Kim, the KoRIA Trial Group, Ji Won Park, Gwonhwa Song, Jiyen Ham, Sunwoo Park, Ga Won Yim, Seung-Hyuk Shim, Beong-Cheol Kang, Suk Joon Chang, Whasun Lim, Jung Chan Lee

**Affiliations:** 1 Department of Obstetrics and Gynecology, Chung-Ang University Hospital, Seoul, 06973 Republic of Korea; 2 Department of Obstetrics and Gynecology, Seoul National University Hospital, 101 Daehak-Ro, Jongno-Gu, Seoul, 03080 Republic of Korea; 3 Department of Obstetrics and Gynecology, Korea University Anam Hospital, Seoul, 02841 Korea; 4 College of Agriculture and Life Sciences, Department of Agricultural Biotechnology, Seoul National University, Seoul, 08826 Republic of Korea; 5 Department of Obstetrics and Gynecology, Seoul National University College of Medicine, Seoul, 03080 Republic of Korea

**Keywords:** Large animal model, Peritoneal metastasis, Immunocompetent, Piglet, Uterine horn

## Abstract

**Background:**

A piglet model for peritoneal metastasis (PM) of ovarian cancer was developed. It will contribute to establishing innovative chemotherapeutical and surgical strategies without any limitation on rodent models.

**Methods:**

A total of 12 four- to five-week-old piglets of 7 to 8 kg were used. Two phases of ovarian cancer cell injections were performed with laparoscopic surgery.

In phase I trial, 5.0 × 10^6^ SK-OV-3 cells in 0.1 ml suspension were inoculated into the omentum, peritoneum, and uterine horns of two piglets twice with a one-week interval. In the phase II trial, 5.0 × 10^6^ SNU-008 cells in 0.1 ml suspension were injected only into uterine horns within the same time frame because tumor implantation after inoculation of SK-OV-3 cells was not observed at the omentum or peritoneum in the phase I trial. Modified peritoneal cancer index (PCI) score was used to monitor tumorigenesis up to 4 weeks after inoculation. Tumor tissues disseminated in the peritoneum 4 weeks after injection were used for histological examination with hematoxylin and eosin (H&E) and paired-box gene 8 (PAX-8) staining.

**Results:**

In the phase I trial, two piglets showed PM with modified PCI scores of 5 and 4 at 3 weeks after the first inoculation, which increased to 14 and 15 after 4 weeks, respectively. In the phase II trial, PM was detected in eight of ten piglets, which showed modified PCI scores of 6 to 12 at 4 weeks after the first inoculation. The overall incidence of PM from the total of 12 piglets after inoculation was 75%. Immunohistochemical H&E and PAX-8 staining confirmed metastatic tumors.

**Conclusions:**

This study provides strong evidence that piglets can be employed as a model for PM by inoculating ovarian cancer cell lines from humans. Using two cell lines, the PM rate is 75%.

**Supplementary Information:**

The online version contains supplementary material available at 10.1186/s12967-022-03533-1.

## Background

Peritoneal metastasis (PM) is characterized by diffuse deposits of tumors on the peritoneal surface. It occurs in up to 75% of patients with solid tumors, including ovarian, colorectal, and gastric cancers [1, 2]. PM-associated solid tumors generally have a poor prognosis with a high mortality rate. They are usually accompanied by increased drug resistance [3, 4]. Thus, new strategies to manage PM-accompanied solid tumors with preclinical and clinical trials and related R&D infrastructure are urgently needed.

Considering animal models for undertaking preclinical studies, only rodent models using immune-deficient mice are available for various PM studies [5, 6]. Immunodeficient rodent models have been used in most cases of experimentation because of their relatively low costs, high success rates of xenograft, ease of handling, and rapid reproduction rates [7, 8]. However, these model systems have apparent limitations, especially for applying the results of such systems to human studies. It is challenging to directly compare tumor response and disease progression between immunodeficient mice and humans. The tiny abdominal cavity of a mouse is another limitation of using a mouse as a model for surgery [9, 10]. Thus, the demand for establishing medium-sized or large animal models to screen pathophysiologic characteristics before human trials are increasing.

The pig is a strong candidate for a mid-sized animal model due to its anatomical and physiological similarities with humans [11–13]. In particular, this species yields a number of advantages such as the similar size of the abdominal cavity, human-like immune system, functional equivalence of multiple diseases with humans, and the feasibility of imaging studies with validated scoring for a target disease. Moreover, it may be possible to avoid artificial manipulation to reduce immune rejection after xenotransplantation of human-derived cells and tissues to some extent. In addition, it might have better ethical acceptance than primates [6, 14–16]. By establishing a mid-sized or large animal model for PM, it will become more feasible to evaluate patterns of metastasis, the effect and safety of anti-cancer drugs, and the feasibility of surgical techniques and medical devices to reduce tumor burdens than by using a rodent model.

Thus, the objective of this study was to develop an immunocompetent piglet model for PM that could be used to xenograft human cancer cells without any treatments to reduce immune reactions. Based on preliminary experiments, the dynamics of PM of ovarian cancer cells were monitored for up to 4 weeks after human cancer cells were xenografted, followed by histological examinations.

## Materials and methods

### Selection of animals and housing

**Fig. 1:**
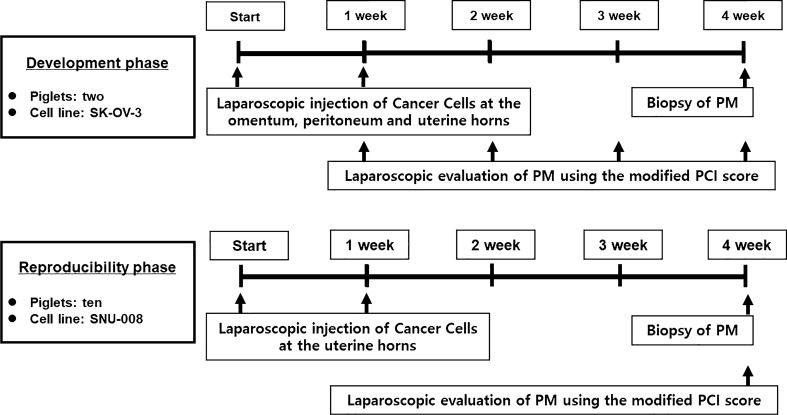
The study protocol for making immunocompetent piglets with peritoneal metastasis using ovarian cancer cell lines

This study was approved by the Institutional Animal Care and Use Committee of Seoul National University Hospital before study initiation (Approval No. 18-0051-S1A0). A total of 12 crossbred piglets among Landrace, Large White, and Duroc breeds at 4 to 5 weeks old with bodyweights of 7 to 8 kg were purchased. Two protocols of xenograft injection were employed. Of those 12 piglets, two were assigned to phase I, and the rest were used for phase II trials. For xenograft inoculation of ovarian cancer cells, piglets were starved for 2 h and administered cefazolin (25 mg/kg; subcutaneous) prophylactically at 30 min before anaesthesia twice every eight hours after surgery. General anaesthesia was conducted using tiletamine/zolazepam (2 mg/kg; intramuscular) and xylazine (2 mg/kg; intramuscular). Isoflurane 2–3% gas was used to maintain anaesthesia during surgery. Postoperative pain was controlled by meloxicam (0.4 mg/kg; subcutaneous; every 24 h). Body weight was evaluated weekly. Finally, all piglets were euthanized with potassium chloride at 4 weeks after surgery for evaluating PM according to the study protocol (Fig. 1).

### Cell lines

For xenograft injection of human ovarian cancer cells, SK-OV-3 cells for phase I and SNU-008 cells for phase II trials were purchased from Koran Cell Line Bank. Each cell line was cultured in Mccoy’s 5a medium (Welgene, Gyeongsan, South Korea) supplemented with 10% heat-inactivated fetal bovine serum (FBS, Welgene) and 1% penicillin/streptomycin (Gibco, Gaithersburg, USA) at 37 °C in a 5% CO_2_ atmosphere. These cells were collected in the exponential phase and digested into single-cell suspensions. The concentration of the suspension of each cell line was then adjusted to 5.0 × 10^6^ cells/0.1 ml.

### Surgical approaches of phase I trial

In the phase I trial, two piglets were inoculated with SK-OV-3 cells. In detail, each piglet was laid down in a Trendelenburg position after general anaesthesia. CO_2_ insufflation was performed for the capnoperitoneum via a Veress needle. Two or three 5-mm bladeless trocars (Eagleport^®^; Dalim Medical Corp., Seoul, South Korea) were then inserted along the midline of the abdomen, which was used as a passage for inserting laparoscopic devices (KARL STORZ Endoscopy Korea CO., Ltd., South Korea).

**Fig. 2:**
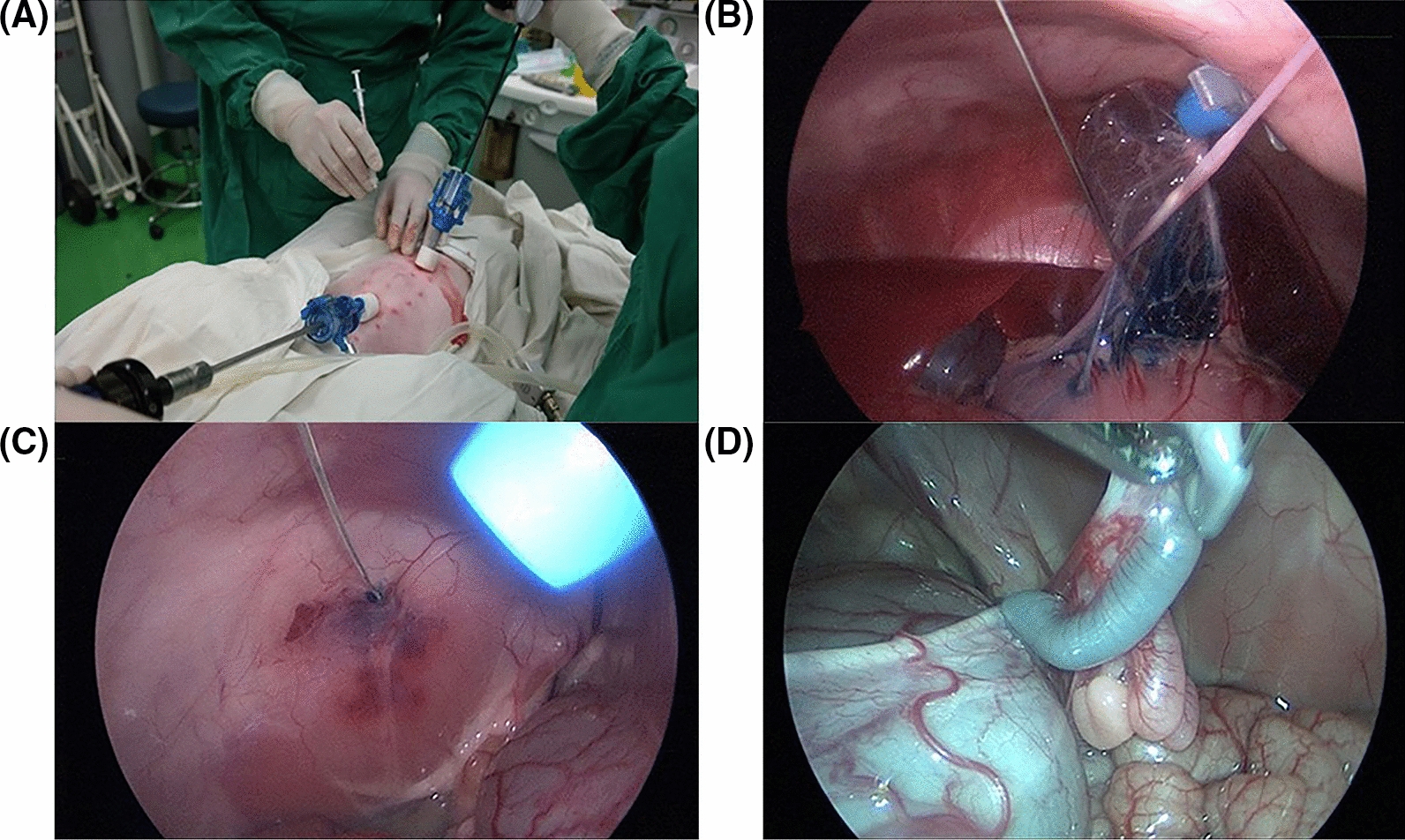
Inoculation of human ovarian cancer cells by laparoscopic surgery into immunocompetent piglets: **A** Each 0.1 ml of SK-OV-3 cell line suspension (5.0 × 10^6^ cells/0.1 ml) was injected percutaneously with a 24G spinal needle at the following five injection sites: **B** fat tissues in the omentum; **C** submesothelial tissues of right and left peritoneum; **D** the uterine cavity within right and left uterine horns

Thereafter, five vials of 0.1 ml of SK-OV-3 cell line suspension were prepared. Each suspension was mixed with a 0.1% solution of indigo carmine to discriminate the injection site. Five injection sites were determined as follows: fat tissues of the omentum; submesothelial tissues of the right and left peritoneum; and the uterine cavity within the right and left uterine horns. Every 0.1 ml of SK-OV-3 cell line suspension was injected with a 24G spinal needle into each injection site (Fig. 2). This inoculation was repeated at the same injection site after 1 week to increase the success rate of PM.

**Fig. 3:**
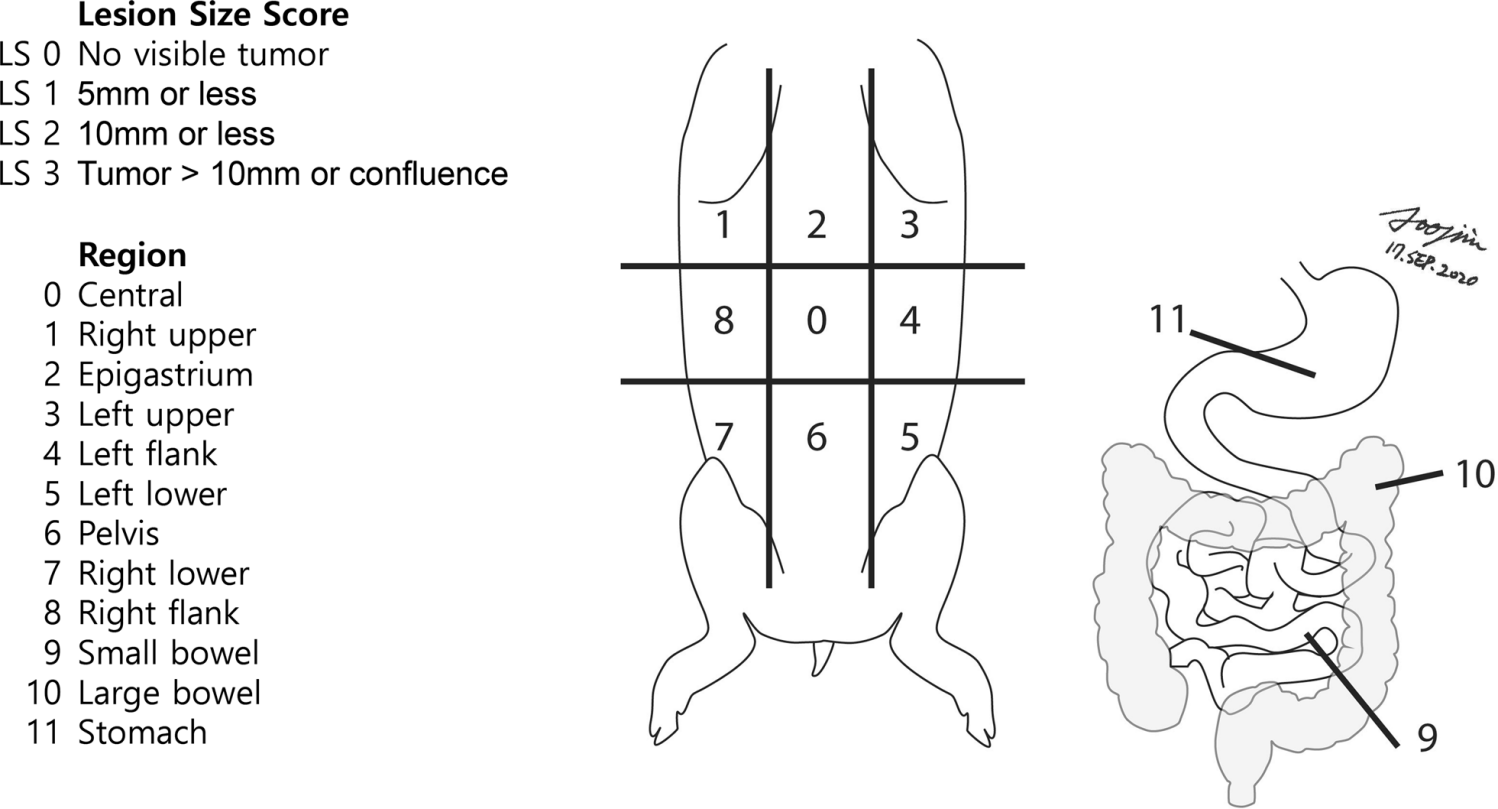
Modified Peritoneal Cancer Index (PCI) in piglets

After xenograft injection of human ovarian cancer cells, the implantation of cancer cells and the pattern of progression every week were evaluated using laparoscopy. To determine the implantation of cancer cells, biopsies were performed for suspicious lesions for histopathologic confirmation. To evaluate the pattern of progression, a modified peritoneal cancer index (PCI) was used based on PCI for patients with PM [17]. The modified PCI included nine parietal regions (central, right upper, epigastrium, left upper, left flank, left lower, pelvis, right lower, and right flank regions) and three visceral regions (small bowel, large bowel, and stomach). The score in each region was calculated according to the lesion size as follows: score of 0, no visible tumor; score of 1, 5 mm or less; score of 2, 6–10 mm; and score of 3, > 10 mm or confluent. The severity of PM was evaluated using total score of all regions (Fig. 3).

### Surgical approaches of the phase II trial

After successful production of large animal models with PM in the phase I trial, the production process of immunocompetent large animal model with PM was then applied to a phase II trial. First, ten piglets were inoculated with SNU-008 cells according to the same surgical procedure. In particular, SNU-008 cells were only injected within the uterine cavity of the right and left uterine horns because tumor implantation after inoculation of SK-OV-3 cells was not observed at the omentum or the peritoneum in the phase I trial (Additional file 1: Video S1). This inoculation was repeated at the same injection site after 1 week to increase the success rate of PM. The implantation of cancer cells and the pattern of progression were evaluated by performing biopsies for suspicious lesions and evaluating modified PCI scores after sacrificing all piglets 4 weeks after surgery.

### Histopathology and immunohistochemistry

After tissues, including tumors disseminated in the peritoneum, were obtained from piglets, all tissues were fixed in 4% paraformaldehyde, dehydrated, paraffin-embedded, and sectioned (6–8 μm in thickness). All sections were stained with hematoxylin and eosin (H&E) to identify tumor cells. Moreover, paired-box gene 8 (PAX8) staining was performed to determine if tumor cells originated from human high-grade serous ovarian carcinoma [18, 19].

## Results

### Immunocompetent piglets with peritoneal metastasis were made by inoculation of SK-OV-3 cells in the phase I trial

**Fig. 4:**
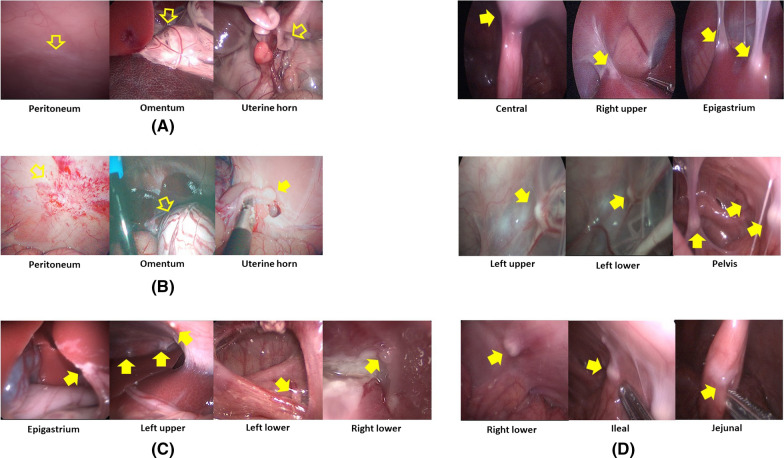
Progression of peritoneal metastasis after inoculation of SK-OV-3 cells into the first piglet during the development phase: **A** Only whitish scars at the injection sites after 1 week; **B** Swelling of the uterine horn adhered to the adjacent peritoneum after 2 weeks; **C** Metastatic tumors developed in the epigastrium, left upper, left lower, and right lower regions after 3 weeks; **D** Metastatic tumors developed in the central, right upper, epigastrium, left upper, left lower, pelvis, right lower, ileal, and jejunal regions after 4 weeks

In the phase I trial employing two piglets, implantation and dissemination of SK-OV-3 cells were observed by laparoscopy. In the first piglet, only whitish scars at the injection sites were observed after 1 week. After 2 weeks, swelling of the uterine horn was observed. It adhered to the adjacent peritoneum despite no suspicious lesions of tumor implantation at the omentum or the peritoneum. After 3 weeks, the first piglet showed metastatic tumors in the epigastrium, left upper, left lower, and right lower regions with a modified PCI score of 5. After 4 weeks, it showed metastatic tumors in the central, right upper, epigastrium, left upper, left lower, pelvis, right lower, ileal, and jejunal regions with a modified PCI score of 14 (Fig. 4).

**Fig. 5:**
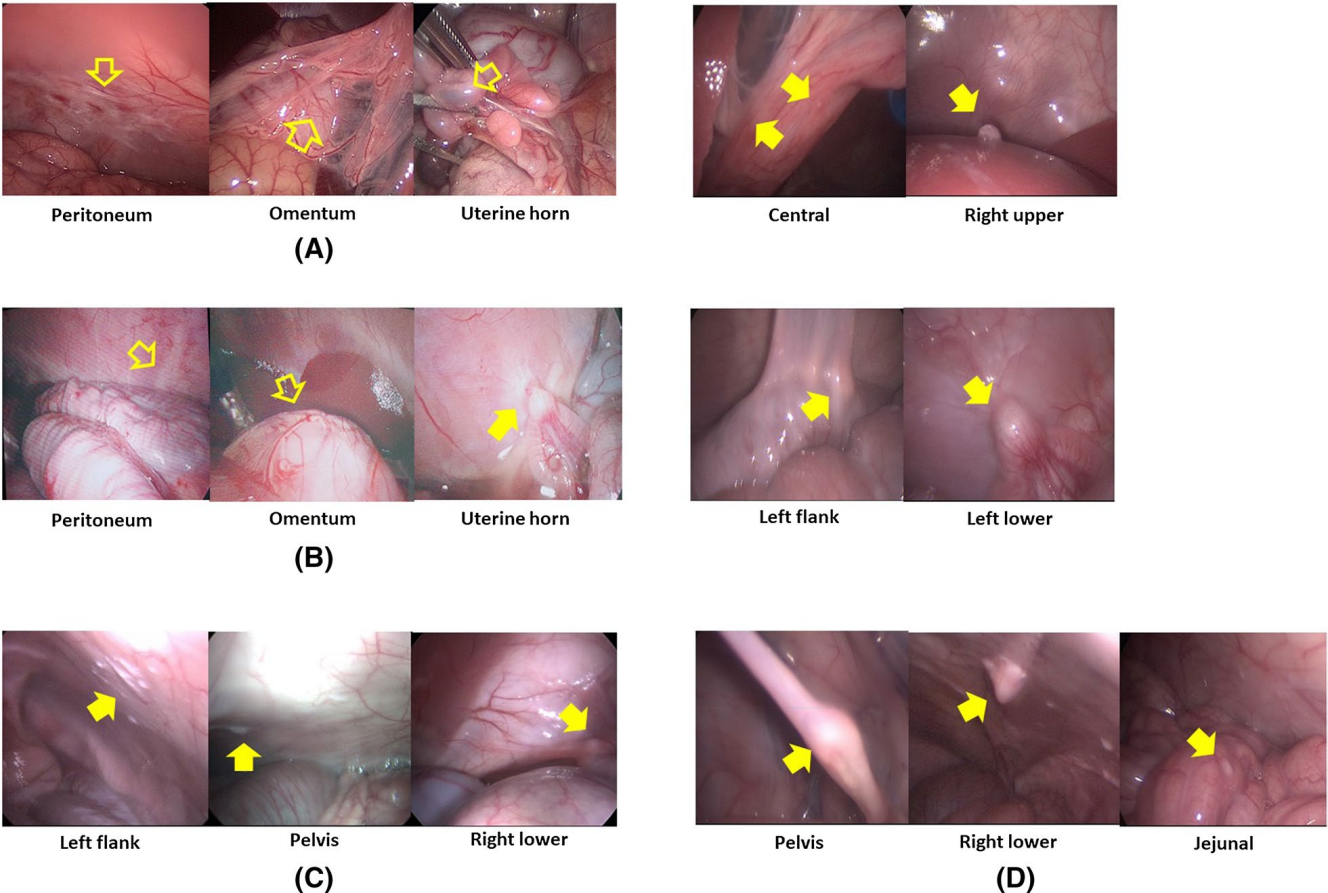
Progression of peritoneal metastasis after inoculation of SK-OV-3 cells into the second piglet during the development phase: **A** No metastatic lesions after 1 week; **B** swelling of the uterine horn adhered to the adjacent peritoneum after 2 weeks; **C** metastatic tumors developed in the left flank, pelvis, and right lower regions after 3 weeks; **D** metastatic tumors developed in the central, right upper, left flank, left lower, pelvis, right lower, and jejunal regions after 4 weeks

In the second piglet, the injected fluid still remained in the uterine horn, whereas there were no metastatic lesions except only whitish scars at the injection sites after 1 week. Swelling of the uterine horn that adhered to the adjacent peritoneum was also found, although there were no suspicious lesions of tumor implantation at the omentum or the peritoneum after 2 weeks. After 3 weeks, the second piglet showed metastatic lesions in the left flank, pelvis, and right lower regions with a modified PCI score of 4. After 4 weeks, it demonstrated metastatic lesions in the central, right upper, left flank, left lower, pelvis, right lower, and jejunal regions with a modified PCI score of 15 (Fig. 5 and Table 1).

**Table 1:** The modified peritoneal cancer index score 3 and 4 weeks after inoculation of SK-OV-3 cells in the development phase

Regions	1st piglet		2nd piglet	
	After 3 weeks	After 4 weeks	After 3 weeks	After 4 weeks
Central	0	2	0	2
Right upper	0	1	0	0
Epigastrium	1	2	0	0
Left upper	1	1	0	1
Left flank	0	0	1	2
Left lower	2	1	0	2
Pelvis	0	1	1	2
Right lower	1	3	2	2
Right flank	0	0	0	0
Small bowel	0	2	0	2
Large bowel	0	0	0	2
Stomach	0	1	0	0
Total	5	14	4	15

### Immunocompetent piglets with peritoneal metastasis were also made by inoculation of SNU-008 cells in the phase II trial

**Fig. 6:**
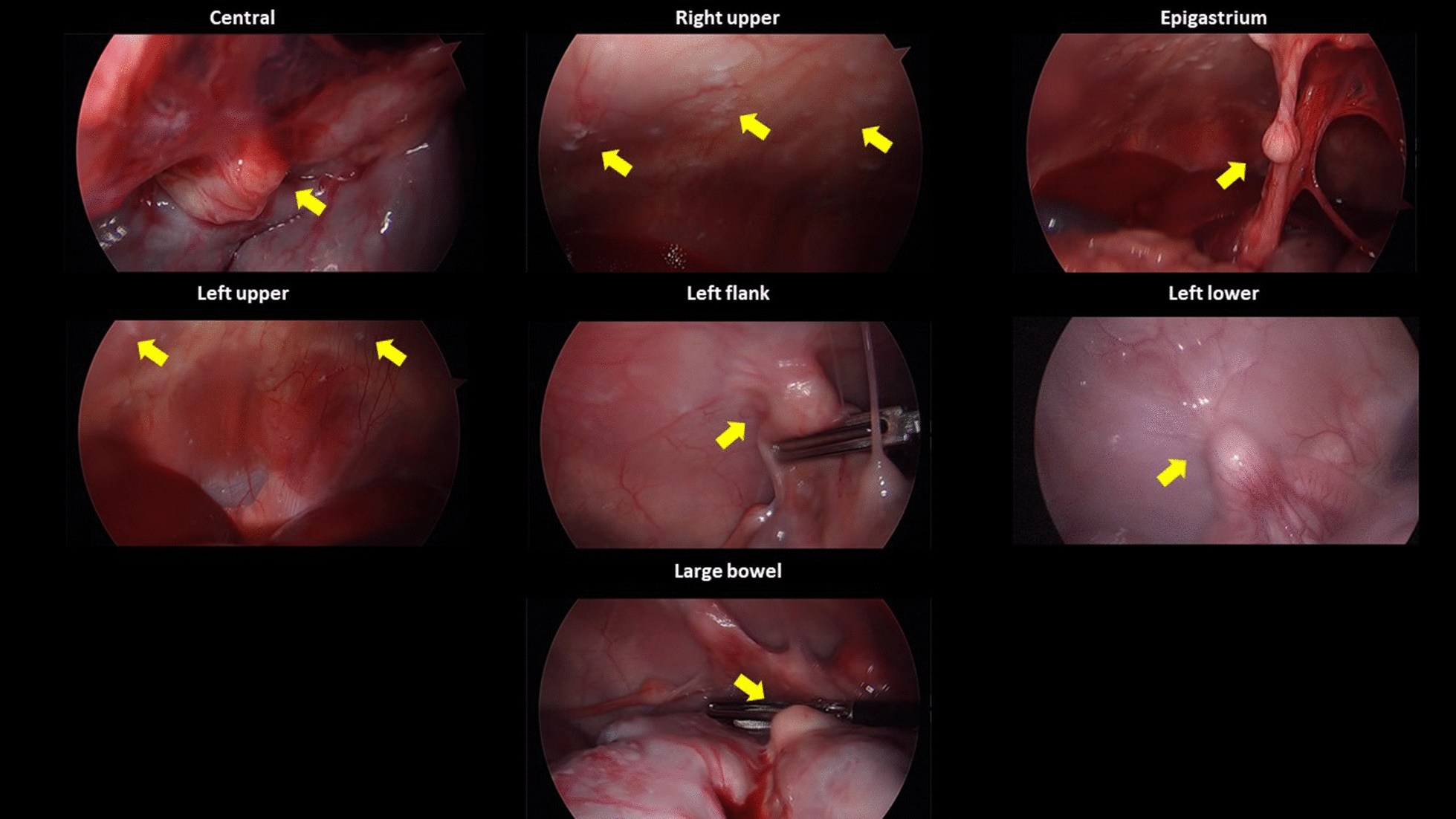
Progression of peritoneal metastasis after inoculation of SNU-008 cells into the first piglet during the reproducibility phase. Metastatic tumors were developed in the central, right upper, epigastrium, left upper, left flank, left lower, and large bowel regions after 4 weeks

**Fig. 7:**
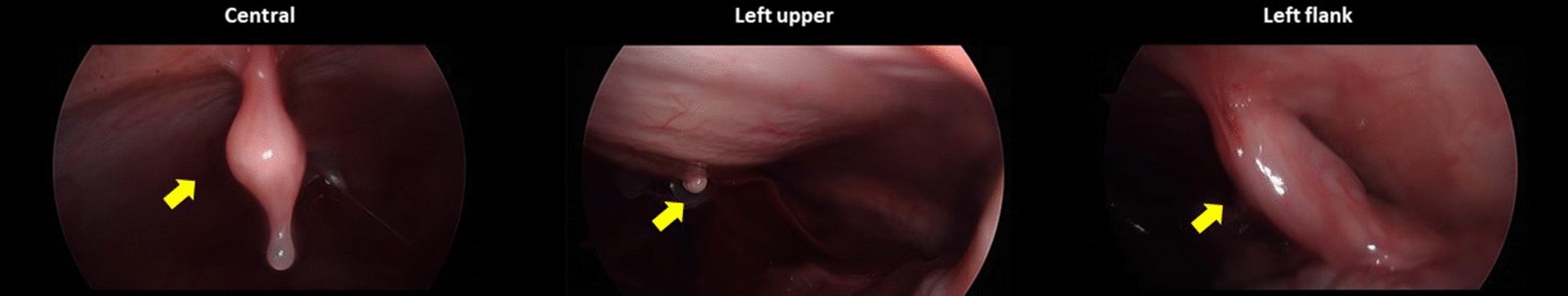
Progression of peritoneal metastasis after inoculation of SNU-008 cells into the second piglet during the reproducibility phase. Metastatic tumors were developed in the central, left upper, and left flank regions after 4 weeks

**Fig. 8:**
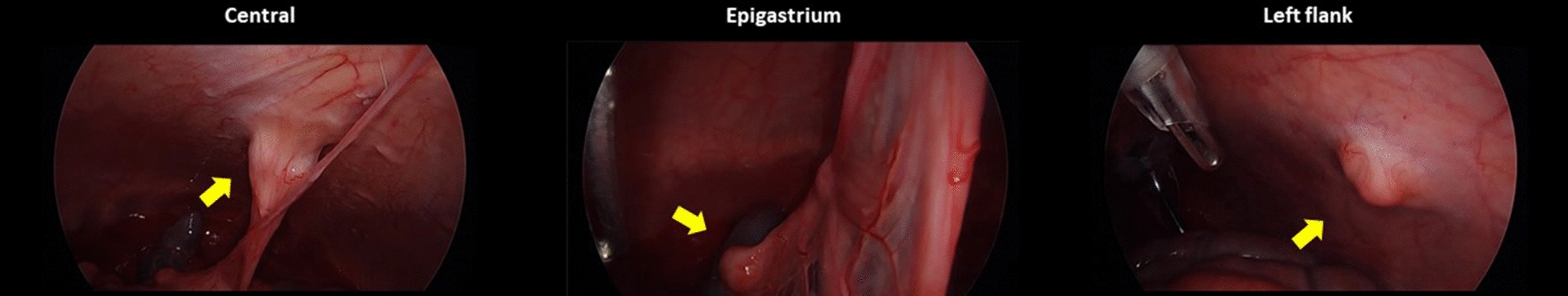
Progression of peritoneal metastasis after inoculation of SNU-008 cells into the third piglet during the reproducibility phase. Metastatic tumors were developed in the central and epigastrium and left flank regions after 4 weeks

**Fig. 9:**
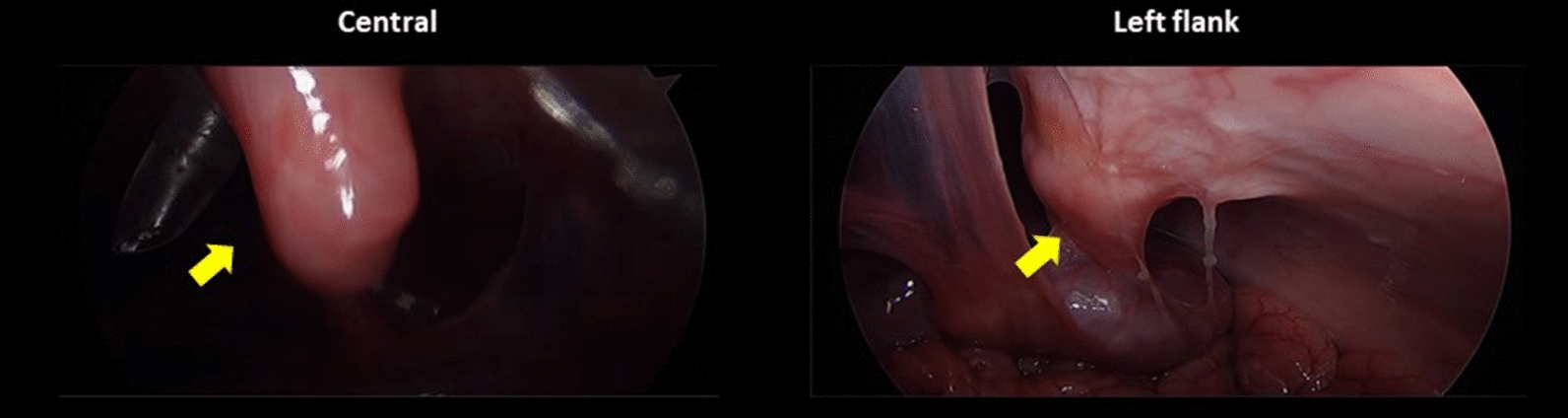
Progression of peritoneal metastasis after inoculation of SNU-008 cells into the fourth piglet during the reproducibility phase. Metastatic tumors were developed in the central and left flank regions after 4 weeks

**Fig. 10:**
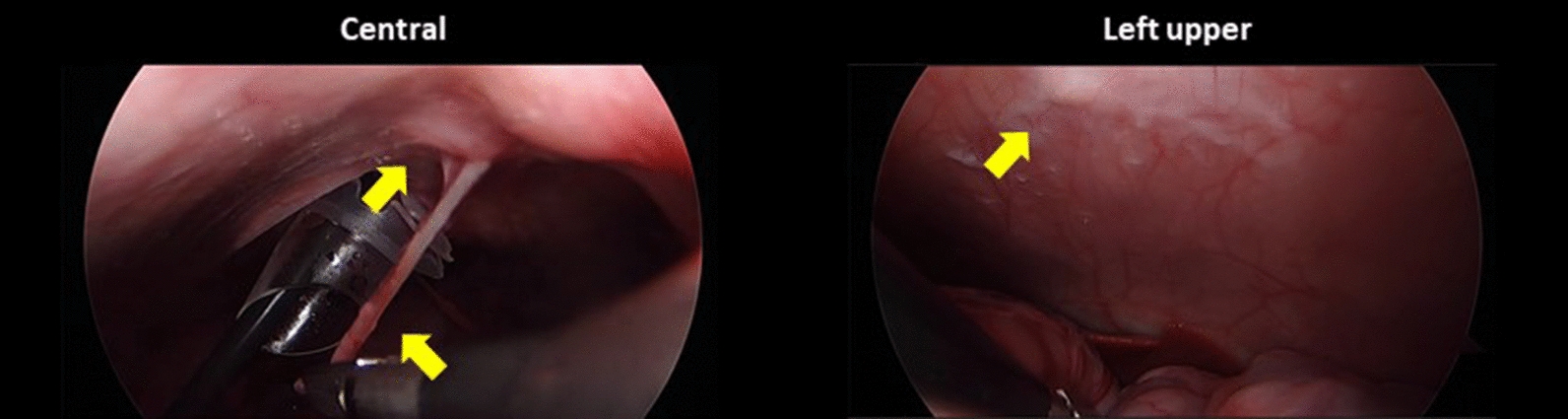
Progression of peritoneal metastasis after inoculation of SNU-008 cells into the fifth piglet during the reproducibility phase. Metastatic tumors were developed in the central and left flank regions after 4 weeks

**Fig. 11:**
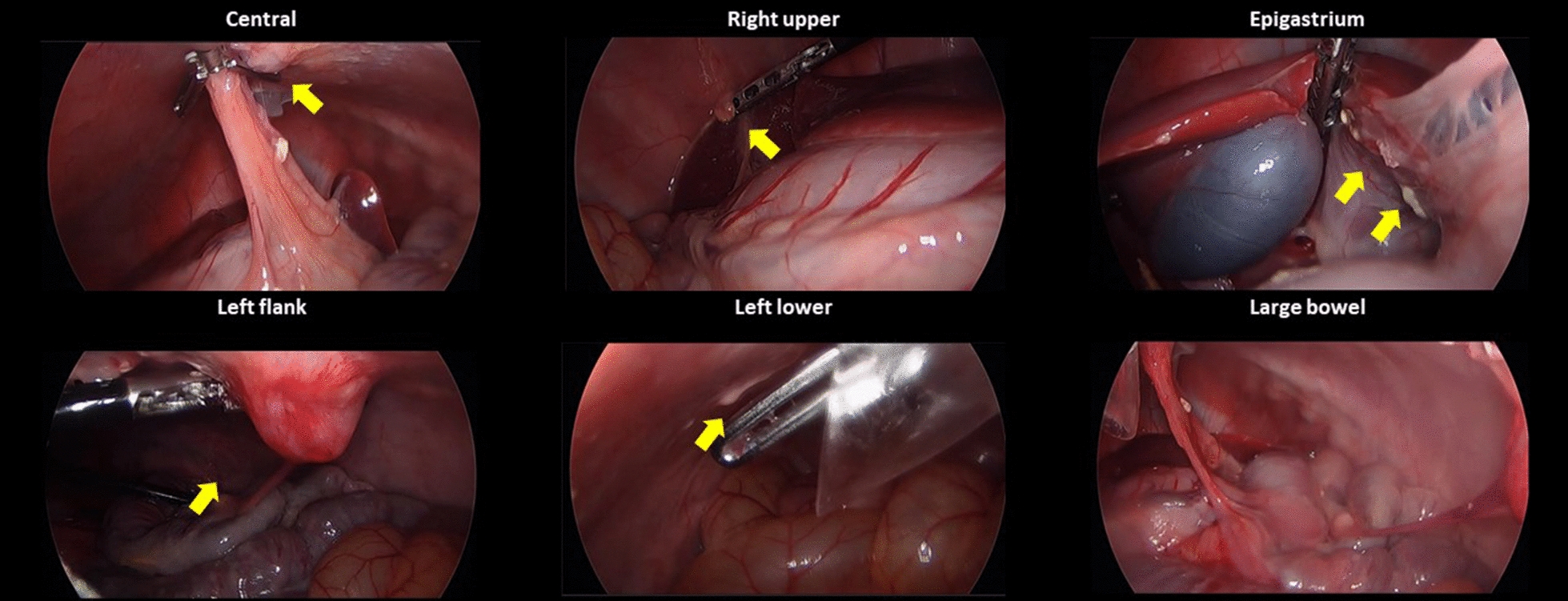
Progression of peritoneal metastasis after inoculation of SNU-008 cells into the eighth piglet during the reproducibility phase. Metastatic tumors were developed in the central, right upper, epigastrium, left flank, left lower, and large bowel regions after 4 weeks

**Fig. 12:**
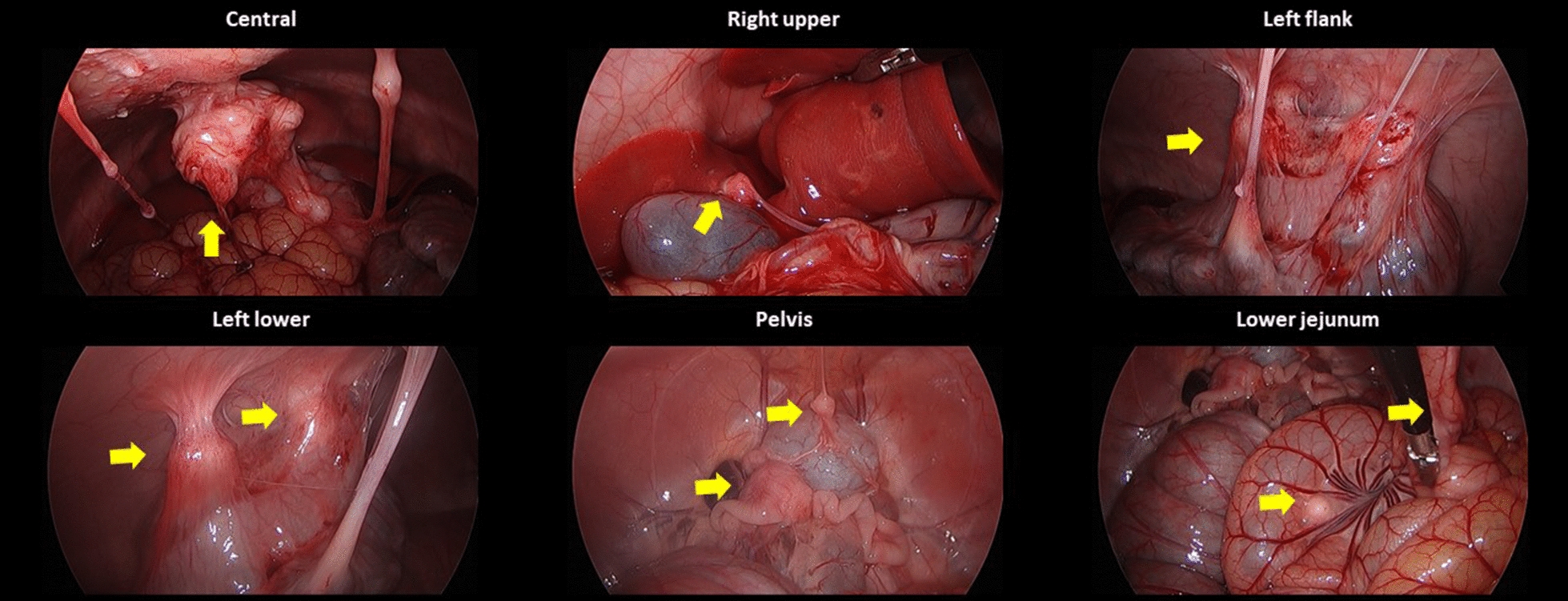
Progression of peritoneal metastasis after inoculation of SNU-008 cells into the tenth piglet during the reproducibility phase. Metastatic tumors were developed in the central, right upper, left flank, left lower, pelvis, and small bowel regions after 4 weeks

Ten immunocompetent piglets were inoculated with SNU-008 cells. Of those, PM was developed in eight piglets. The modified PCI score ranged from two to 15 (Figs. 6, 7, 8, 9, 10, 11, 12 and Table 2). The success rate of producing an immunocompetent large animal model with PM was 100% (2/2) after inoculation of SK-OV-3 cells and 70% (7/10) after inoculation of SNU-008 cells, with an overall success rate of 75% (9/12).

**Table 2:** The modified peritoneal cancer index score 4 weeks after inoculation of SNU-008 cells in the reproducibility phase

Regions	1st piglet	2nd piglet	3rd piglet	4th piglet	5th piglet	6th piglet	7th piglet	8th piglet	9th piglet	10th piglet
Central	3	2	2	3	1	0	0	3	0	3
Right upper	1	0	0	0	0	0	0	1	0	2
Epigastrium	2	0	1	0	0	0	0	1	0	0
Left upper	1	1	0	0	1	0	0	0	0	0
Left flank	3	2	2	3	0	0	0	3	0	3
Left lower	3	0	0	0	0	0	0	1	0	3
Pelvis	0	0	0	0	0	0	0	0	0	2
Right lower	0	0	0	0	0	0	0	0	0	0
Right flank	0	0	0	0	0	0	0	0	0	0
Small bowel	0	0	0	0	0	0	0	0	0	2
Large bowel	1	0	0	0	0	0	0	1	0	0
Stomach	0	0	0	0	0	0	0	0	0	0
Total	14	5	5	6	2	0	0	10	0	15

### Metastatic tumor cells were seen in H&E and PAX8 staining

**Fig. 13:**
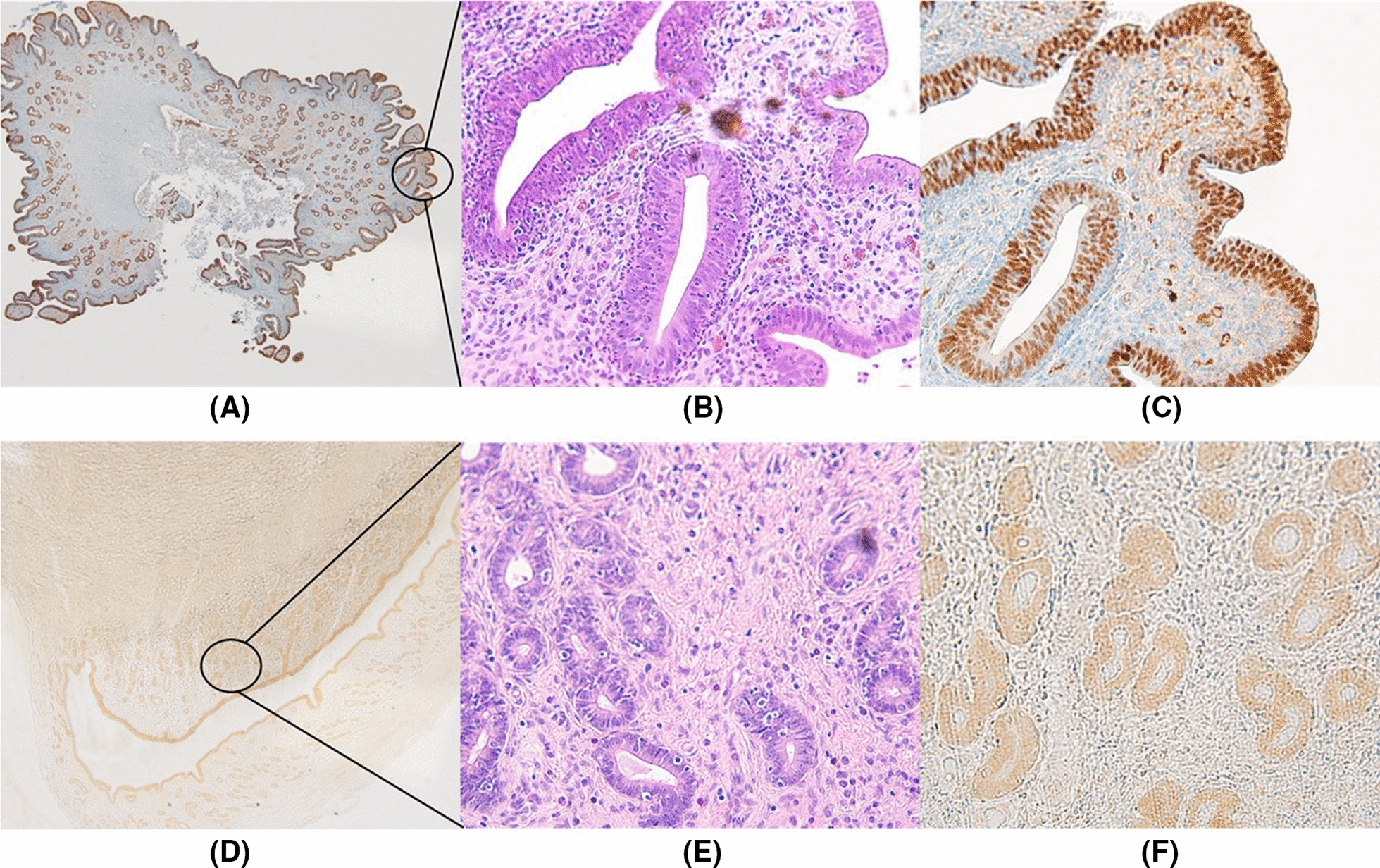
Pathological identification of metastatic peritoneal tumors: **A** Metastatic tumors after inoculation of SK-OV-3 cells (× 5) on **B** hematoxylin and eosin (H&E) staining and **C** paired-box gene 8 (PAX8) staining (× 400); **C** Metastatic tumor after inoculation of SNU-008 cells (× 5) on **D** H&E and **E** PAX8 staining (× 400)

On immunohistochemical H&E staining, metastatic tumors obtained from piglets with PM were pathologically identified. Moreover, tumor cells were stained by PAX8, suggesting human high-grade serous ovarian carcinoma (Fig. 13).

## Discussion

Since animal models in which epithelial cells undergo neoplastic transformation can provide opportunities to evaluate cellular and molecular changes related to malignant transformation, immunodeficient rodent models have been used to create PM using cancer cells or tissue xenografts due to their cost-effectiveness [20]. However, these models are too small to evaluate the efficacy or safety of new methods such as intraperitoneal chemotherapy, hyperthermic intraperitoneal chemotherapy (HIPEC), pressurized intraperitoneal aerosol chemotherapy (PIPAC), and precision surgery that can selectively remove tumor cells expressing specific biomarkers for treating PM [21–23].

To the best of our knowledge, this is the first study to succeed in making an immunocompetent mid-sized animal model with PM. We secured the manufacturing technology required to fabricate this animal model successfully. We employed commercially available crossbred pigs for this experimentation. Xenograft injection of human cancer cells into pigs was possible without showing any immunological complications up to 4 weeks after inoculation. The piglet model system suggested in this study can improve the efficacy due to the use of commercially available species and modeling without any immunologic treatments to increase the feasibility. In preclinical studies using this mid-size animal model, we can evaluate tumor responses after HIPEC or PIPAC medical devices according to the PCI applied to the human body. Moreover, cytoreductive surgery can be conducted to remove tumors that have metastasized to the abdominopelvic peritoneum and visceral organs of sizes similar to those of the human body.

Up to now, many studies have attempted to develop a PM model in different species. A rabbit PM model for intraperitoneal tumors was developed in a previous study, where the greater omentum and the upper abdominal cavity of rabbits weighing from 2.5 kg to 3 kg, success rates of PM formation were 100% (12/12), 91.7% (11/12), and 58.3% (7/12), respectively after VX2 gastric cancer cells were injected into the submucosal layer of the stomach [24]. Nevertheless, there is a limitation in that this model is still too small to be applied for surgery or intraperitoneal chemotherapy. Attempts have also been made in pigs. After HeLa cells were injected into the abdominal cavity with laparoscopic surgery, the success rate of producing intraperitoneal tumors was 63.8% (23/36) on port sites [25]. Furthermore, severe combined immunodeficient (SCID) pigs have been employed for cancer cell xenograft [26, 27]. However, PM was localized to the surgical incision site and subcutaneous or intramuscular layers in these models.

In this study, we developed a piglet PM model using 4- to 5-week-old piglets for acquiring immunocompetence. Although a pig fetus becomes immunocompetent at about 80 days of gestation [28], the piglet’s immune system is not complete till 6 weeks of age because it depends on passive immunity using maternally derived immunoglobulins before weaning of milk [29]. Thus, we used 4 to 5 weeks old piglets with “the window of vulnerability” for making this immunocompetent mid-sized animal model with PM. This could contribute to the success of cancer cell housing after xenotransplantation, while the only limited time of observation period can be possible.

**Fig. 14:**
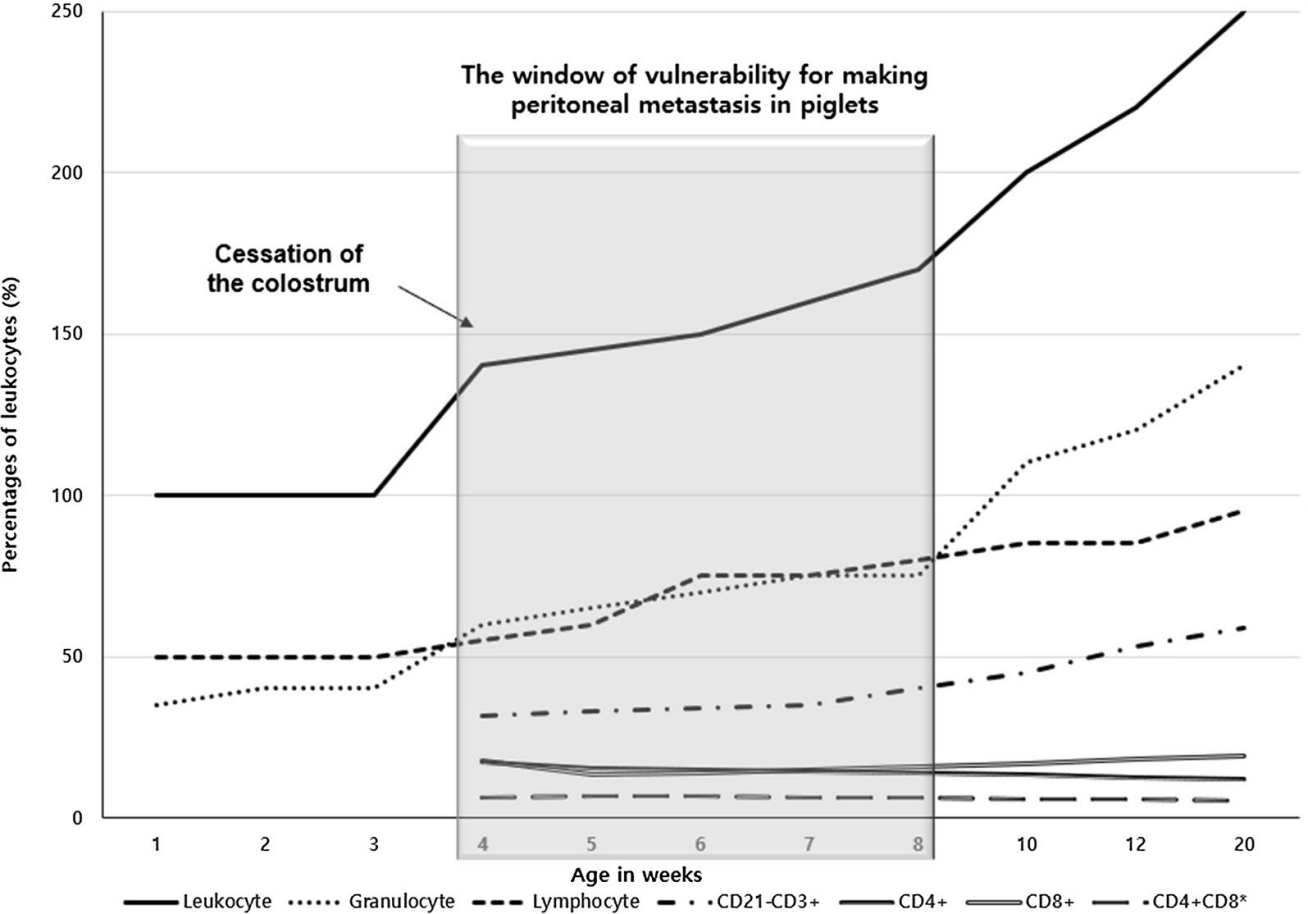
Percentage of leukocytes, granulocytes, lymphocytes, and their subpopulations in the peripheral blood in pigs according to age after birth

In general, leukocytes, granulocytes, and lymphocytes increase after the cessation of colostral intake 3 to 4 weeks after birth based on previous reports where age-dependent immunologic changes in piglets were evaluated [30, 31]. Although lymphocytes and T cells (CD21-CD3 +) increase gradually, granulocytes increase abruptly 8 weeks after birth. Thus, the graduality of lymphocytosis may be important for maximally extending the successful period of cancer cell housing after xenotransplantation, whereas rapid granulocytosis may hinder it by their destructive role of tissue damage by massive releases of oxidative and proteolytic molecules [32]. Thus, “the window of vulnerability” can be considered 4 to 8 weeks after birth for making immunocompetent piglets with PM (Fig. 14).

The repetitive injection of tumor cells into the uterine horn might have contributed to the successful xenograft in this study. Although we injected tumor cells twice into three common tumorigenic sites (the omentum, submesothelial layers of the peritoneum, and the uterine horn), we found that the fluid with tumor cells after the first injection remained in the uterine horn, whereas tumor formation was not confirmed at other sites at the time of the second injection of tumor cells. Finally, we found a 75% success rate in constructing an immunocompetent large animal model with PM and that five out of nine piglets with PM showed metastatic tumors from the uterine horn adhered to the pelvic cavity despite different types of cancer cell lines.

In particular, the uterine horn has the advantage in that tumor cells can be continuously discharged into the abdominopelvic cavity while maintaining the concentration of tumor cells for a long time in a space like a balloon tube. Moreover, the porcine endometrium consists of predominantly stromal and glandular cells secreting prostaglandins E and F_2α_ [33], which can promote tumorigenesis and metastasis by immunosuppression [34].

Nevertheless, this study has some limitations as follows. First, there was no information on whether PM would worsen or become alleviated when active immunity was completed because we sacrificed all 4 to 5 weeks old piglets 4 weeks after this experiment. Since we used “the window of vulnerability” to increase tumor implantation, tumors in this model can be regressed as piglets grow, and active immunity is strengthened. Second, this immunocompetent model has been established in the relative duration of immunosuppression, called “the window of vulnerability,” in piglets. Thus, this model cannot be helpful in immune-oncology agents such as immune checkpoint inhibitors because the immune reaction related to cancer treatment may not be anticipated in this model. Moreover, this model may not help investigate the efficacy of chemotherapeutic or targeted agents 8 weeks after birth because human cancer cells implanted in the peritoneum can be regressed by acquired immunocompetence beyond the window of vulnerability. Third, we used only female piglets and ovarian cancer cell lines for making this model. Thus, further research is needed to make male piglets with PM using different types of cancer cell lines.

## Conclusion

We report the establishment of an immunocompetent piglet (4- to 5-week-old) model for PM that can be used in preclinical studies for evaluating the efficacy and safety of intravenous or intraperitoneal usage of anti-cancer agents in various types of solid tumors with PM. Moreover, this model helps pioneer the field of precision surgery that selectively removes tumor-specific tissues in the era of precision medicine. However, there was a limitation in that only 4 weeks were observed due to the acquisition of immunity with the growth of piglets. Long term-observation of more than four weeks should be made in the future to increase the feasibility of this piglet PM model.

## Supplementary Information

**Additional file 1:** **Video S1.** Injection of human ovarian cancer cells within the right and left uterine horns of the uterine cavity for making piglets with peritoneal metastasis.

## Data Availability

The datasets generated during and/or analyzed during the current study are available from the corresponding author upon reasonable request.

## Abbreviations

H&E: Hematoxylin and eosin
HIPEC: Hyperthermic intraperitoneal chemotherapy
PAX8: Paired-box gene 8
PCI: Peritoneal cancer index
PIPAC: Pressurized intraperitoneal aerosol chemotherapy
PM: Peritoneal metastasis
SCID: Severe combined immunodeficient

## References

[1] Schorge JO, McCann C, Del Carmen MG (2010). Surgical debulking of ovarian cancer: what difference does it make?. Rev Obstet Gynecol.

[2] Coccolini F, Gheza F, Lotti M, Virzi S, Iusco D, Ghermandi C (2013). Peritoneal carcinomatosis. World J Gastroenterol.

[3] Glehen O, Mohamed F, Gilly FN (2004). Peritoneal carcinomatosis from digestive tract cancer: new management by cytoreductive surgery and intraperitoneal chemohyperthermia. Lancet Oncol.

[4] Chu DZ, Lang NP, Thompson C, Osteen PK, Westbrook KC (1989). Peritoneal carcinomatosis in nongynecologic malignancy. A prospective study of prognostic factors. Cancer.

[5] Akhter J, Yao P, Johnson LA, Riordan SM, Morris DL (2008). A new peritoneal carcinomatosis model in cyclosporine immunosuppressed rats. Anticancer Res.

[6] Gerdts V, Wilson HL, Meurens F, van Drunen Littel-van den S, Wilson D, Walker S (2015). Large animal models for vaccine development and testing. ILAR J.

[7] Ziegler A, Gonzalez L, Blikslager A (2016). Large animal models: the key to translational discovery in digestive disease research. Cell Mol Gastroenterol Hepatol.

[8] Kobaek-Larsen M, Thorup I, Diederichsen A, Fenger C, Hoitinga MR (2000). Review of colorectal cancer and its metastases in rodent models: comparative aspects with those in humans. Comp Med.

[9] Boivin GP, Washington K, Yang K, Ward JM, Pretlow TP, Russell R (2003). Pathology of mouse models of intestinal cancer: consensus report and recommendations. Gastroenterology.

[10] Flisikowska T, Merkl C, Landmann M, Eser S, Rezaei N, Cui X (2012). A porcine model of familial adenomatous polyposis. Gastroenterology.

[11] Vodicka P, Smetana K, Dvorankova B, Emerick T, Xu YZ, Ourednik J (2005). The miniature pig as an animal model in biomedical research. Ann N Y Acad Sci.

[12] Meurens F, Summerfield A, Nauwynck H, Saif L, Gerdts V (2012). The pig: a model for human infectious diseases. Trends Microbiol.

[13] Henze LJ, Koehl NJ, O'Shea JP, Kostewicz ES, Holm R, Griffin BT (2019). The pig as a preclinical model for predicting oral bioavailability and in vivo performance of pharmaceutical oral dosage forms: a PEARRL review. J Pharm Pharmacol.

[14] Ribitsch I, Baptista PM, Lange-Consiglio A, Melotti L, Patruno M, Jenner F (2020). Large animal models in regenerative medicine and tissue engineering: to do or not to do. Front Bioeng Biotechnol.

[15] Aigner B, Renner S, Kessler B, Klymiuk N, Kurome M, Wunsch A (2010). Transgenic pigs as models for translational biomedical research. J Mol Med (Berl).

[16] Casal M, Haskins M (2006). Large animal models and gene therapy. Eur J Hum Genet.

[17] Jacquet P, Sugarbaker PH (1996). Clinical research methodologies in diagnosis and staging of patients with peritoneal carcinomatosis. Cancer Treat Res.

[18] Xiang L, Kong B (2013). PAX8 is a novel marker for differentiating between various types of tumor, particularly ovarian epithelial carcinomas. Oncol Lett.

[19] Chai HJ, Ren Q, Fan Q, Ye L, Du GY, Du HW (2017). PAX8 is a potential marker for the diagnosis of primary epithelial ovarian cancer. Oncol Lett.

[20] Vanderhyden BC, Shaw TJ, Ethier JF (2003). Animal models of ovarian cancer. Reprod Biol Endocrinol.

[21] van Driel WJ, Koole SN, Sonke GS (2018). Hyperthermic intraperitoneal chemotherapy in ovarian cancer. N Engl J Med.

[22] Alyami M, Hubner M, Grass F, Bakrin N, Villeneuve L, Laplace N (2019). Pressurised intraperitoneal aerosol chemotherapy: rationale, evidence, and potential indications. Lancet Oncol.

[23] van Dam GM, Themelis G, Crane LM, Harlaar NJ, Pleijhuis RG, Kelder W (2011). Intraoperative tumor-specific fluorescence imaging in ovarian cancer by folate receptor-alpha targeting: first in-human results. Nat Med.

[24] Mei LJ, Yang XJ, Tang L, Hassan AH, Yonemura Y, Li Y (2010). Establishment and identification of a rabbit model of peritoneal carcinomatosis from gastric cancer. BMC Cancer.

[25] Reymond MA, Tannapfel A, Schneider C, Scheidbach H, Kover S, Jung A (2000). Description of an intraperitoneal tumour xenograft survival model in the pig. Eur J Surg Oncol.

[26] Boettcher AN, Kiupel M, Adur MK, Cocco E, Santin AD, Bellone S (2019). Human ovarian cancer tumor formation in severe combined immunodeficient (SCID) pigs. Front Oncol.

[27] Boettcher AN, Loving CL, Cunnick JE, Tuggle CK (2018). Development of severe combined immunodeficient (SCID) pig models for translational cancer modeling: future insights on how humanized SCID pigs can improve preclinical cancer research. Front Oncol.

[28] Salmon H (1984). Immunity in the fetus and the newborn infant: a swine model. Reprod Nutr Dev.

[29] Sinkora M, Butler JE (2009). The ontogeny of the porcine immune system. Dev Comp Immunol.

[30] Pietrasina O, Miller J, Rząsa A (2020). Differences in the relative counts of peripheral blood lymphocytes subsets in various age groups of pigs. Can J Vet Res..

[31] Pomorska-Mol M, Markowska-Daniel I (2011). Age-dependent changes in relative and absolute size of lymphocytes subsets in the blood of pigs from birth to slaughter. Bull Vet Inst Pulawy..

[32] Scozzi D, Ibrahim M, Menna C, Krupnick AS, Kreisel D, Gelman AE (2017). The role of neutrophils in transplanted organs. Am J Transplant.

[33] Blackwell DM, Speth RC, Mirando MA (2003). Morphometric analysis of the uterine endometrium of swine on days 12 and 16 postestrus. Anat Rec A Discov Mol Cell Evol Biol.

[34] Wang T, Jing B, Xu D, Liao Y, Song H, Sun B (2020). PTGES/PGE2 signaling links immunosuppression and lung metastasis in Gprc5a-knockout mouse model. Oncogene.

